# Supplementary material for: Evaluating the efficacy of curcumin in the management of oral potentially malignant disorders: a systematic review and meta-analysis
Source: PeerJ. 2024 Nov 15;12:e18492. doi: 10.7717/peerj.18492 (PMC11572357; doi:10.7717/peerj.18492)
Supplement: Supplemental Information 1 [file peerj-12-18492-s001.pdf]

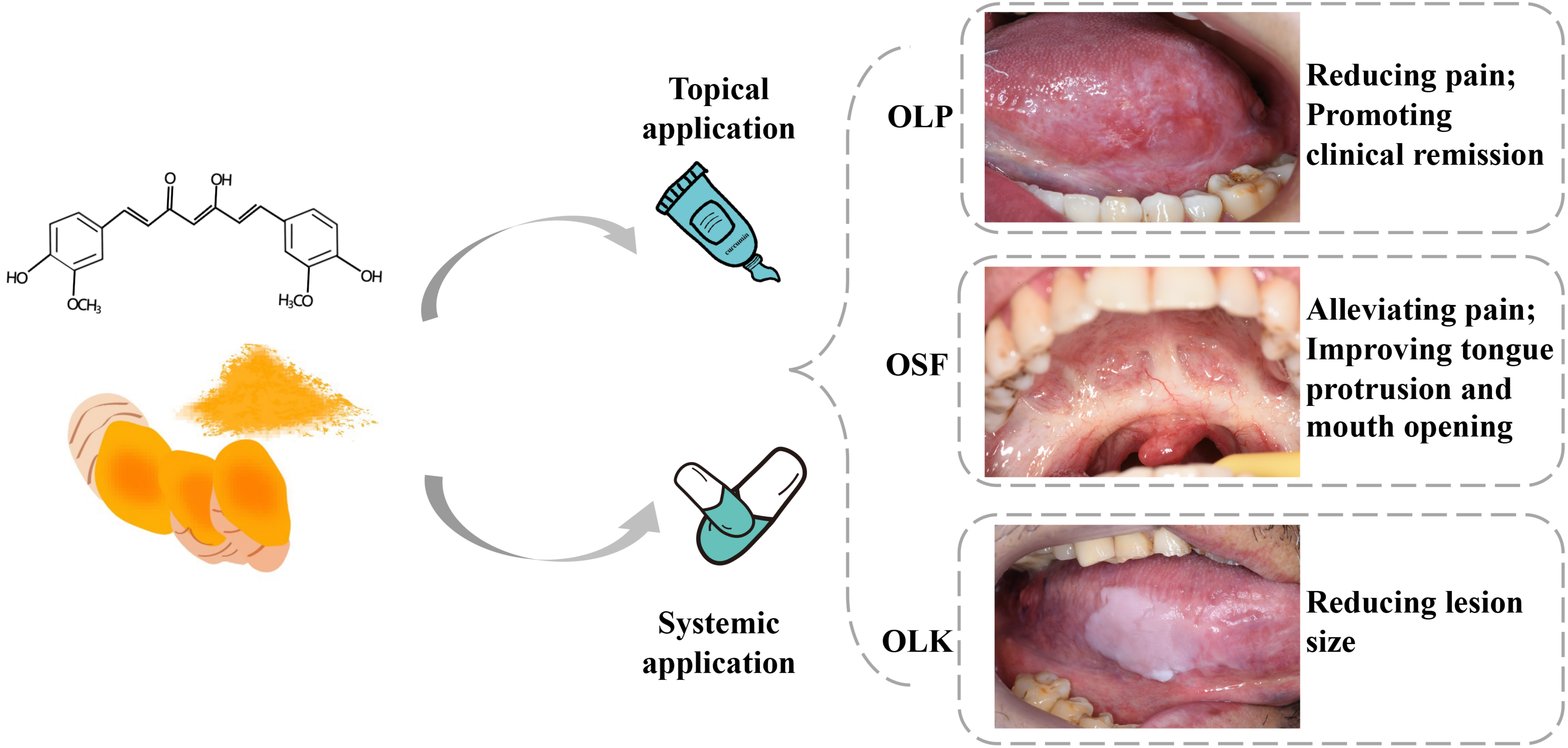
O
OH
HO
OCH3
H3CO
OH
Topical application
curcumin
Systemic application
OLP
OSF
OLK
Reducing pain; Promoting clinical remission
Alleviating pain; Improving tongue protrusion and mouth opening
Reducing lesion size
