# Supplementary material for: Evaluating the efficacy of curcumin in the management of oral potentially malignant disorders: a systematic review and meta-analysis
Source: PeerJ. 2024 Nov 15;12:e18492. doi: 10.7717/peerj.18492 (PMC11572357; doi:10.7717/peerj.18492)
Supplement: Supplemental Information 4 [file peerj-12-18492-s004.docx]

**Table S1: Detailed search strategy for each database.**

|  | **Steps** | **Strategies** |
| --- | --- | --- |
| **PubMed** | #1 | ("Leukoplakia, Oral"[Mesh]) OR "Lichen Planus, Oral"[Mesh]) OR "Oral Submucous Fibrosis"[Mesh]) OR "Lupus Erythematosus, Discoid"[Mesh]) OR "Actinic cheilitis"[Supplementary Concept] |
|  | #2 | ("Curcumin"[Mesh]) OR "Curcuma"[Mesh] |
|  | #3 | "Oral potentially malignant disorders"[All Fields] OR "Oral potentially malignant lesions"[All Fields] OR "Oral precancer"[All Fields] OR " Oral premalignant lesions"[All Fields] OR "Oral submucous fibrosis"[All Fields] OR "Oral leukoplakia"[All Fields] OR "Proliferative verrucous leukoplakia"[All Fields] OR "Oral erythroplakia"[All Fields] OR " Oral lichen planus"[All Fields] OR "Discoid lupus erythematosus"[All Fields] OR "Actinic cheilitis"[All Fields] |
|  | #4 | "Turmeric"[All Fields] OR "Curcumin"[All Fields] OR Curcuma longa"[All Fields] OR " Diferuloylmethane"[All Fields] |
|  | #5 | #1 OR #3 |
|  | #6 | #2 OR #4 |
|  | #7 | #5 AND #6 |
| **Web of Science** | #1 | ALL=(Oral potentially malignant lesions or Oral precancer or Oral premalignant lesions or Oral premalignant lesions or Oral lichen planus or Leukoplakia or Oral submucous fibrosis or Erythroplakia or Oral lichenoid lesions or Proliferative verrucous leukoplakia or Actinic cheilitis ) |
|  | #2 | ALL=(Turmeric or Curcumin or Diferuloylmethane or Turmeric yellow or Curcuma longa) |
|  | #3 | #1 AND #2 |
| **Ovid-Embase** | #1 | (Oral potentially malignant lesions or Oral precancer or Oral premalignant lesions or Oral premalignant lesions or Oral lichen planus or Leukoplakia or Oral submucous fibrosis or Erythroplakia or Oral lichenoid lesions or Proliferative verrucous leukoplakia or Actinic cheilitis ).af. |
|  | #2 | (Turmeric or Curcumin or Diferuloylmethane or Turmeric yellow or Curcuma longa).af. |
|  | #3 | #1 AND #2 |
| **Cochrane Library** | #1 | (Turmeric or Curcumin or Diferuloylmethane or Turmeric yellow or Curcuma longa) and (Oral potentially malignant lesions or Oral precancer or Oral premalignant lesions or Oral premalignant lesions or Oral lichen planus or Leukoplakia or Oral submucous fibrosis or Erythroplakia or Oral lichenoid lesions or Proliferative verrucous leukoplakia or Actinic cheilitis) in All Text |
