# Supplementary material for: Evaluating the efficacy of curcumin in the management of oral potentially malignant disorders: a systematic review and meta-analysis
Source: PeerJ. 2024 Nov 15;12:e18492. doi: 10.7717/peerj.18492 (PMC11572357; doi:10.7717/peerj.18492)
Supplement: Supplemental Information 5 [file peerj-12-18492-s005.docx]

| **Supplementary Table 2: List of excluded studies and according reasons.** | | | |
| --- | --- | --- | --- |
| **#** | **Authors** | **Title** | **Reasons for the exclusion** |
| **1** | Bohra et al. 2021 | Black Turmeric and Aloe Vera in the Management of Oral Submucous Fibrosis: A Prospective Clinical Study | The efficacy of curcumin could not be individually evaluated. |
| **2** | Srivastava et al. 2015 | Clinical evaluation of the role of tulsi and turmeric in the management of oral submucous fibrosis: A pilot, prospective observational study | The efficacy of curcumin could not be individually evaluated, and therapy of control groups contains curcumin or Curcuma longa Extract. |
| **3** | Bakhshi et al. 2020 | Combination Therapy with 1% Nanocurcumin Gel and 0.1% Triamcinolone Acetonide Mouth Rinse for Oral Lichen Planus: A Randomized Double-Blind Placebo Controlled Clinical Trial | The efficacy of curcumin could not be individually evaluated. |
| **4** | Mansourian et al. 2017 | Comparison of the Efficacy of Topical Triamcinolone in Orabase and Curcumin in Orabase in Oral Graft-Versus-Host Disease. | The efficacy of curcumin could not be individually evaluated. |
| **5** | Khaitan et al. 2022 | Curcuma Longa in the Treatment of Symptomatic oral lichen planus: A non-randomized controlled trial | The efficacy of curcumin could not be individually evaluated. |
| **6** | Deepak et al. 2021 | Efficacy of Curcumin and Topical Steroids in the Treatment for Oral Submucous Fibrosis: A Comparative study | The efficacy of curcumin could not be individually evaluated. |
| **7** | Darakhsha et al. 2019 | Efficacy of curcumin gel and tulsi gel in oral submucous fibrosis | The efficacy of curcumin could not be individually evaluated. |
| **8** | Mahato et al. 2019 | Evaluation of Efficacy of Curcumin along with Lycopene and Piperine in the Management of Oral Submucous Fibrosis | The efficacy of curcumin could not be individually evaluated. |
| **9** | Amirchaghi et al. 2016 | Evaluation of the Efficacy of Curcumin in the Treatment of Oral Lichen Planus: A Randomized Controlled Trial | The efficacy of curcumin could not be individually evaluated. |
| **10** | Vishwakar et al. 2018 | Potent Antitumor Effects of a Combination of Three Nutraceutical Compounds | The efficacy of curcumin could not be individually evaluated. |
| **11** | Deb et al. 2022 | Role of nano curcumin on superoxide dismutase levels in leukoplakia | No control group |
| **12** | Singh et al. 2013 | Turmeric - A new treatment option for lichen planus: A pilot study | No control group |
| **13** | Aditi et al. 2021 | A clinicobiochemical evaluation of curcumin as gel and as buccal mucoadhesive patches in the management of oral submucous fibrosis | Therapy of control groups contains curcumin or Curcuma longa Extract. |
| **14** | Hazzah et al. 2016 | A new approach for treatment of precancerous lesions with curcumin solid–lipid nanoparticle-loaded gels: in vitro and clinical evaluation | Therapy of control groups contains curcumin or Curcuma longa Extract. |
| **15** | Gupta et al. 2020 | A novel mixture of curcumin paste and prednisolone for treating oral lichen planus: A case controlled comparative study | Therapy of control groups contains curcumin or Curcuma longa Extract. |
| **16** | Kapoor et al. 2019 | Effect of curcumin in management of potentially malignant disorders-A comparative study. | Therapy of control groups contains curcumin or Curcuma longa Extract. |
| **17** | Hastak et al. 1997 | Effect of turmeric oil and turmeric oleoresin on cytogenetic damage in patients suffering from oral submucous fibrosis | Therapy of control groups contains curcumin or Curcuma longa Extract. |
| **18** | Cheng et al. 2001 | Phase I clinical trial of curcumin, a chemopreventive agent, in patients with high-risk or pre-malignant lesions | Therapy of control groups contains curcumin or Curcuma longa Extract. |
| **19** | Rai et al. 2010 | Possible action mechanism for curcumin in pre-cancerous lesions based on serum and salivary markers of oxidative stress | Therapy of control groups contains curcumin or Curcuma longa Extract. |
| **20** | Chainani et al. 2012 | Use of curcuminoids in a cohort of patients with oral lichen planus, an autoimmune disease | Therapy of control groups contains curcumin or Curcuma longa Extract. |
| **21** | Chainani et al. 2008 | Validation of instruments to measure the symptoms and signs of oral lichen planus | Incomplete outcome indicators |
| **22** | Chainani et al. 2007 | A randomized, placebo-controlled, double-blind clinical trial of curcuminoids in oral lichen planus | Incomplete outcome indicators |
| **23** | Singh et al. 2013 | Turmeric - A new treatment option for lichen planus – a pilot study | Incomplete outcome indicators |
| **24** | Mehta et al. 2020 | Assessing the Effect of Curcumin on the Oral Mucosal Cytomorphometry and Candidal Species Specificity in Tobacco Users: A Pilot Study | In vitro study |
| **25** | Sterniczuk et al. 2022 | Effectiveness of Curcumin in Reducing Self-Rated Pain-Levels in the Orofacial Region: A Systematic Review of Randomized-Controlled Trials | Systematic review |
| **26** | Neetha et al. 2020 | Chemopreventive Synergism between Green Tea Extract and Curcumin in Patients with Potentially Malignant Oral Disorders: A Double-blind, Randomized Preliminary Study | Participants were patients with OPMDs，while the specific OPMD was not clarified. |
| **27** | Pipalia et al. 2016 | Clinicobiochemical evaluation of turmeric with black pepper and nigella sativa in management of oral submucous fibrosis—a double-blind, randomized preliminary study | The efficacy of curcumin could not be evaluated solely. |
| **28** | Joshi et al. 2003 | Early human safety study of turmeric oil (Curcuma longa oil) administered orally in healthy volunteers | Participants were volunteers rather than people with OPMDs. |
| **29** | Ghobadi et al. 2021 | Effect of Curcumin on Oral Lichen Planus: A Single Blind Randomized Controlled Clinical Triall. | The language is not English. |
| **30** | Chainani et al. 2012 | High-dose curcuminoids are efficacious in the reduction in symptoms and signs of oral lichen planus. | OLP was only diagnosed clinically. |
| **31** | Kopuri et al. 2016 | A comparative study of the clinical efficacy of lycopene and curcumin in the treatment of oral submucous fibrosis using ultrasonography. | The full text is not accessible. |
| **32** | Das et al. 2010 | Comparative Study of the Efficacy of Curcumin and Turmeric Oil as Chemopreventive Agents in Oral Submucous Fibrosis: A Clinical and Histopathological Evaluation. | Incomplete outcome indicators |
| **33** | Agarwal et al. 2014 | Evaluation of efficacy of turmeric in management of oral submucous fibrosis. | The full text is not accessible. |
| **34** | Mobeen et al. 2023 | A Novel Herbal Paste Formulation of Turmeric, Tulsi, and Honey for the Treatment of Oral Submucous Fibrosis. | The efficacy of curcumin could not be individually evaluated. |
| **35** | Tepan et al. 2023 | Efficacy of Combination of Curcumin-Piperine with Antioxidants in Oral Submucous Fibrosis: A Randomized, Open-Label Study. | The efficacy of curcumin could not be individually evaluated. |

**References:**

1. Bohra A, Maheswari TNU, Harsh A, Garg A. Black Turmeric and Aloe Vera in the Management of Oral Submucous Fibrosis: A Prospective Clinical Study. Asian Pac J Cancer Prev. 2021 Dec 1;22(12):3941-3947. doi: 10.31557/APJCP.2021.22.12.3941.

2. Srivastava A, Agarwal R, Chaturvedi TP, Chandra A, Singh OP. Clinical evaluation of the role of tulsi and turmeric in the management of oral submucous fibrosis: A pilot, prospective observational study. J Ayurveda Integr Med. 2015 Jan-Mar;6(1):45-9. doi: 10.4103/0975-9476.146563.

3. Bakhshi M, Gholami S, Mahboubi A, Jaafari MR, Namdari M. Combination Therapy with 1% Nanocurcumin Gel and 0.1% Triamcinolone Acetonide Mouth Rinse for Oral Lichen Planus: A Randomized Double-Blind Placebo Controlled Clinical Trial. Dermatol Res Pract. 2020 May 20;2020:4298193. doi: 10.1155/2020/4298193.

4. Mansourian A, Bahar B, Moosavi MS, Amanlou M, Babaeifard S. Comparison of the Efficacy of Topical Triamcinolone in Orabase and Curcumin in Orabase in Oral Graft-Versus-Host Disease. J Dent (Tehran). 2017 Nov;14(6):313-320.

5. Khaitan T, Vishal, Kabiraj A, Sinha DK, Ranjan R, Singh R. Curcuma Longa in the Treatment of Symptomatic oral lichen planus: A non-randomized controlled trial. Indian J Dermatol. 2022 Jul-Aug;67(4):478. doi: 10.4103/ijd.ijd_1065_20.

6. Deepak TA, Manjunath M, Thakur H. Efficacy of Curcumin and Topical Steroids in the Treatment for Oral Submucous Fibrosis: A Comparative study. J Pharm Bioallied Sci. 2021 Jun;13(Suppl 1):S542-S548. doi: 10.4103/jpbs.JPBS_687_20.

7. Rizvi D , Abidi A , Faiz S , et al. Efficacy of curcumin gel and tulsi gel in oral submucous fibrosis[J]. National Journal of Physiology Pharmacy and Pharmacology, 2019:1.

8. Mahato B, Prodhan C, Mandal S, Dutta A, Kumar P, Deb T, Jha T, Chaudhuri K. Evaluation of Efficacy of Curcumin along with Lycopene and Piperine in the Management of Oral Submucous Fibrosis. Contemp Clin Dent. 2019 Jul-Sep;10(3):531-541. doi: 10.4103/ccd.ccd_937_18.

9. Amirchaghmaghi M, Pakfetrat A, Delavarian Z, Ghalavani H, Ghazi A. Evaluation of the Efficacy of Curcumin in the Treatment of Oral Lichen Planus: A Randomized Controlled Trial. J Clin Diagn Res. 2016 May;10(5):ZC134-7. doi: 10.7860/JCDR/2016/16338.7870.

10. Vishwakarma V, New J, Kumar D, Snyder V, Arnold L, Nissen E, Hu Q, Cheng N, Miller D, Thomas AR, Shnayder Y, Kakarala K, Tsue TT, Girod DA, Thomas SM. Potent Antitumor Effects of a Combination of Three Nutraceutical Compounds. Sci Rep. 2018 Aug 15;8(1):12163. doi: 10.1038/s41598-018-29683-1.

11. Deb S, Bhargava D, Bansal P, Kanuru V. Role of nano curcumin on superoxide dismutase levels in leukoplakia. J Oral Maxillofac Pathol. 2022 Jan-Mar;26(1):21-25. doi: 10.4103/jomfp.JOMFP_267_21.

12. Singh V, Pal M, Gupta S, Tiwari SK, Malkunje L, Das S. Turmeric - A new treatment option for lichen planus: A pilot study. Natl J Maxillofac Surg. 2013 Jul;4(2):198-201. doi: 10.4103/0975-5950.127651.

13. Thimmasetty J. A clinicobiochemical evaluation of curcumin as gel and as buccal mucoadhesive patches in the management of oral submucous fibrosis. Oral Surg Oral Med Oral Pathol Oral Radiol. 2021 Apr;131(4):428-434. doi: 10.1016/j.oooo.2020.12.020.

14. Hazzah HA, Farid RM, Nasra MM, Zakaria M, Gawish Y, El-Massik MA, Abdallah OY. A new approach for treatment of precancerous lesions with curcumin solid-lipid nanoparticle-loaded gels: in vitro and clinical evaluation. Drug Deliv. 2016 May;23(4):1409-19. doi: 10.3109/10717544.2015.1065524.

15. Naik S R , Gupta P , Khaitan T , et al. A Novel Mixture of Curcumin Paste and Prednisolone for Treating Oral Lichen Planus: A Case Controlled Comparative Study. 2020.

16. Kapoor S., Arora P. Effect of curcumin in management of potentially malignant disorders-A comparative study. Onkol. Radioter. 2019;46(1):1-4.

17. Hastak K, Lubri N, Jakhi SD, More C, John A, Ghaisas SD, Bhide SV. Effect of turmeric oil and turmeric oleoresin on cytogenetic damage in patients suffering from oral submucous fibrosis. Cancer Lett. 1997 Jun 24;116(2):265-9. doi: 10.1016/s0304-3835(97)00205-x.

18. Cheng AL, Hsu CH, Lin JK, Hsu MM, Ho YF, Shen TS, Ko JY, Lin JT, Lin BR, Ming-Shiang W, Yu HS, Jee SH, Chen GS, Chen TM, Chen CA, Lai MK, Pu YS, Pan MH, Wang YJ, Tsai CC, Hsieh CY. Phase I clinical trial of curcumin, a chemopreventive agent, in patients with high-risk or pre-malignant lesions. Anticancer Res. 2001 Jul-Aug;21(4B):2895-900.

19. Rai B, Kaur J, Jacobs R, Singh J. Possible action mechanism for curcumin in pre-cancerous lesions based on serum and salivary markers of oxidative stress. J Oral Sci. 2010 Jun;52(2):251-6. doi: 10.2334/josnusd.52.251.

20. Chainani-Wu N, Collins K, Silverman S Jr. Use of curcuminoids in a cohort of patients with oral lichen planus, an autoimmune disease. Phytomedicine. 2012 Mar 15;19(5):418-23. doi: 10.1016/j.phymed.2011.11.005.

21. Chainani-Wu N, Silverman S Jr, Reingold A, Bostrom A, Lozada-Nur F, Weintraub J. Validation of instruments to measure the symptoms and signs of oral lichen planus. Oral Surg Oral Med Oral Pathol Oral Radiol Endod. 2008 Jan;105(1):51-8. doi: 10.1016/j.tripleo.2007.06.022.

22. Chainani-Wu N, Silverman S Jr, Reingold A, Bostrom A, Mc Culloch C, Lozada-Nur F, Weintraub J. A randomized, placebo-controlled, double-blind clinical trial of curcuminoids in oral lichen planus. Phytomedicine. 2007 Aug;14(7-8):437-46.

23. Singh V, Pal M, Gupta S, Tiwari SK, Malkunje L, Das S. Turmeric - A new treatment option for lichen planus: A pilot study. Natl J Maxillofac Surg. 2013 Jul;4(2):198-201.

24. Mehta P, Bhavasar R, Ajith NA, Bhavsar RP, Bahammam MA, Bakri MMH, Alzahrani KJ, Alghamdi AA, Halawani IF, Bhandi S, Raj AT, Patil S. Assessing the Effect of Curcumin on the Oral Mucosal Cytomorphometry and Candidal Species Specificity in Tobacco Users: A Pilot Study. Healthcare (Basel). 2022 Aug 10;10(8):1507.

25. Sterniczuk B, Rossouw PE, Michelogiannakis D, Javed F. Effectiveness of Curcumin in Reducing Self-Rated Pain-Levels in the Orofacial Region: A Systematic Review of Randomized-Controlled Trials. Int J Environ Res Public Health. 2022 May 25;19(11):6443.

26. Neetha MC, Panchaksharappa MG, Pattabhiramasastry S, Shivaprasad NV, Venkatesh UG. Chemopreventive Synergism between Green Tea Extract and Curcumin in Patients with Potentially Malignant Oral Disorders: A Double-blind, Randomized Preliminary Study. J Contemp Dent Pract. 2020 May 1;21(5):521-531.

27. Pipalia PR, Annigeri RG, Mehta R. Clinicobiochemical evaluation of turmeric with black pepper and nigella sativa in management of oral submucous fibrosis-a double-blind, randomized preliminary study. Oral Surg Oral Med Oral Pathol Oral Radiol. 2016 Dec;122(6):705-712.

28. Joshi J, Ghaisas S, Vaidya A, Vaidya R, Kamat DV, Bhagwat AN, Bhide S. Early human safety study of turmeric oil (Curcuma longa oil) administered orally in healthy volunteers. J Assoc Physicians India. 2003 Nov;51:1055-60.

29. Nazanin Ghobadi, Simin Lesan, Mandana Khatibi. Effect of Curcumin on Oral Lichen Planus: A Single Blind Randomized Controlled Clinical Triall. J Mazandaran Univ Med Sci 2022; 32 (211): 37-48 (Persian).

30. Chainani-Wu N, Madden E, Lozada-Nur F, Silverman S Jr. High-dose curcuminoids are efficacious in the reduction in symptoms and signs of oral lichen planus. J Am Acad Dermatol. 2012 May;66(5):752-60.

31. Kopuri R K C , Chakravarthy C , Sunder S ,et al.Comparative Study of the Clinical Efficacy of Lycopene and Curcumin in the Treatment of Oral Submucous Fibrosis using Ultrasonography[J]. J Int Oral Health 2016;8:687‑91.

32. Das A, Balan A, Sreelatha K. Comparative study of the efficacy of curcumin and turmeric oil as chemopreventive agents in oral submucous fibrosis: A clinical and histopathological evaluation. J Indian Acad Oral Med Radiol 2010;22:88.

33. Agarwal N, Prasad R, Singh D, Sinha A, Srivastava S, Singh G. Evaluation of efficacy of turmeric in management of oral submucous fibrosis. J Indian Acad Oral Med Radiol 2014;26:260.

34. Mobeen S, SV R, JD S, et al. (October 06, 2023) A Novel Herbal Paste Formulation of Turmeric, Tulsi, and Honey for the Treatment of Oral Submucous Fibrosis. Cureus 15(10): e46608. doi:10.7759/cureus.46608.

35. Tepan M, Patil A, Hebbale M, Agarwal R, Sabane A, Gachake A. Efficacy of Combination of Curcumin–Piperine with Antioxidants in Oral Submucous Fibrosis: A Randomized, Open-Label Study. Journal of Indian Academy of Oral Medicine & Radiology 35(2):p 161-165, Apr–Jun 2023.
