# Supplementary figures and images for: Evaluating the efficacy of curcumin in the management of oral potentially malignant disorders: a systematic review and meta-analysis
Source: PeerJ. 2024 Nov 15;12:e18492. doi: 10.7717/peerj.18492 (PMC11572357; doi:10.7717/peerj.18492)

A

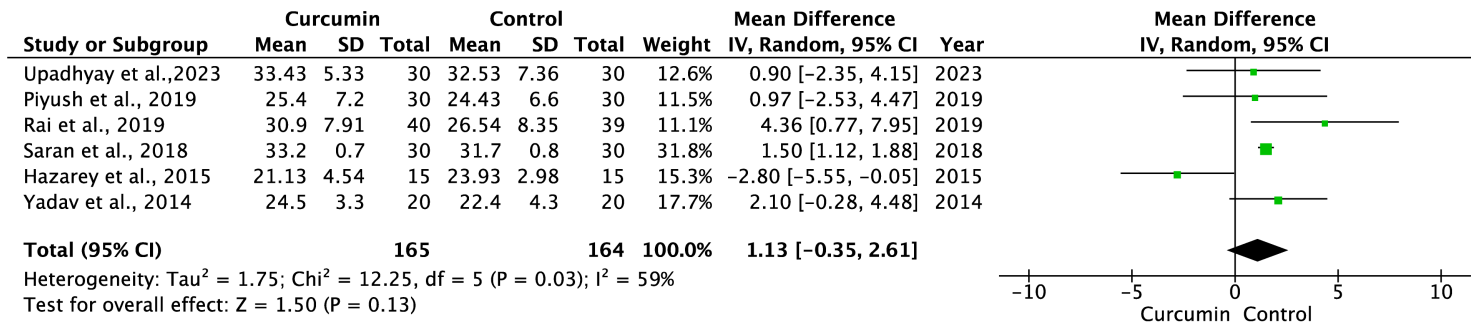

B

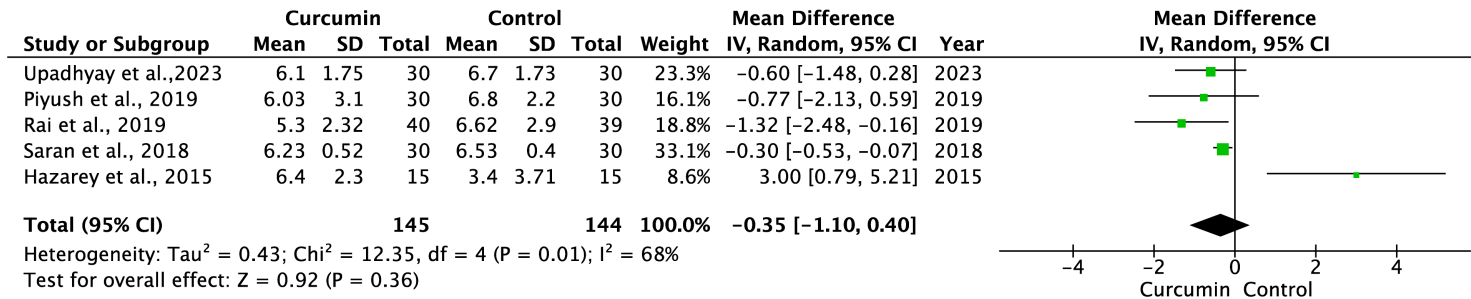

C

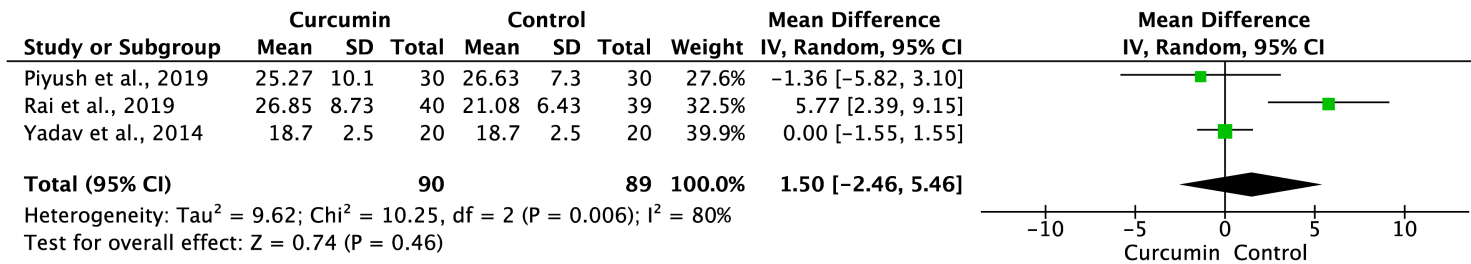

Supplement: Supplemental Information 6 [file peerj-12-18492-s006.pdf]

A

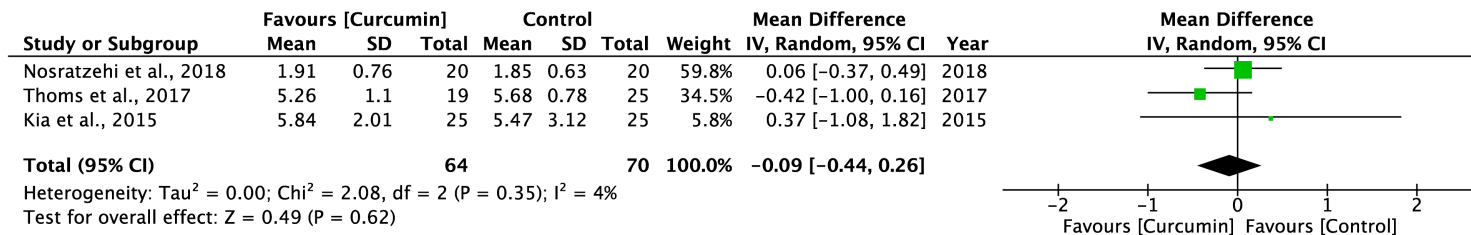

B

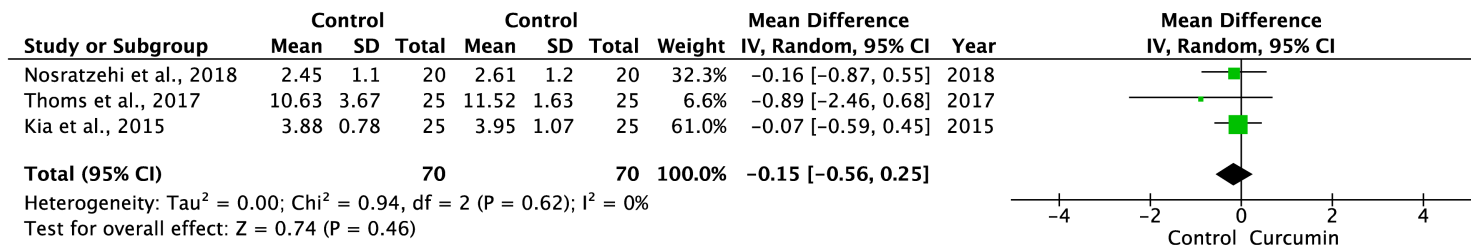

Supplement: Supplemental Information 7 [file peerj-12-18492-s007.pdf]
